# Physical Fitness Characteristics That Relate to Work Sample Test Battery Performance in Law Enforcement Recruits

**DOI:** 10.3390/ijerph15112477

**Published:** 2018-11-06

**Authors:** Robert G. Lockie, J. Jay Dawes, Katherine Balfany, Ciara E. Gonzales, Maria M. Beitzel, Joseph M. Dulla, Robin M. Orr

**Affiliations:** 1 Department of Kinesiology, California State University, Fullerton, CA 92831, USA; kbalfany@csu.fullerton.edu (K.B.); ciaraelena95@csu.fullerton.edu (C.E.G.); mariabeitzel@csu.fullerton.edu (M.M.B.); 2 Department of Health Sciences, University of Colorado-Colorado Springs, Colorado Springs, CO 80918, USA; jdawes@uccs.edu; 3 Recruit Training Unit, Training Bureau, Los Angeles County Sheriff’s Department, Los Angeles, CA 90022, USA; JMDulla@lasd.org; 4 Tactical Research Unit, Bond University, Robina, QLD 4226, Australia; rorr@bond.edu.au

**Keywords:** aerobic capacity, anaerobic capacity, deputy sheriff, job-specific, police, muscular endurance, tactical

## Abstract

This study determined relationships between an agency-specific fitness test battery (PT500), and a work sample test battery (WSTB) in law enforcement recruits. Retrospective analysis on 219 males and 34 females from one agency was conducted. The PT500 comprised: push-ups, sit-ups, and mountain climbers in 120 s; pull-ups; and 201 m and 2.4 km runs. The WSTB comprised: 99 yard (90.53 m) obstacle course (99OC); body drag (BD) with a 165 pound (75 kg) dummy; 6 foot (1.83 m) chain link fence (CLF) and solid wall (SW) climb; and 500 yard (457.2 m) run (500R). Partial correlations, controlling for sex, calculated PT500 and WSTB relationships (*p* < 0.05). Stepwise regression determined whether fitness predicted WSTB performance. The 500R related to all PT500 assessments (*r* range = −0.127–0.574), 99OC related to all bar push-ups and mountain climbers, and BD related to none. The CLF related to sit-ups, pull-ups, and 2.4 km run; SW related to mountain climbers, pull-ups, and 2.4 km run (*r* range = −0.127–−0.315). Push-ups, pull-ups, and 2.4 km run were involved in predictive relationships for 99OC, CLF, SW, and 500R (*r*^2^ range = 0.217–0.500). To perform better in the WSTB and job-specific tasks, developing upper-body strength and aerobic fitness may be beneficial.

## 1. Introduction

Law enforcement is a demanding profession and can place great deal of physical stress on those employed in this vocation. For example, on-duty law enforcement officers (LEOs) may be required to push, pull, lift, carry, or drag objects or people at any time during their shift [1]. In addition to these physical demands, LEOs must also perform job-specific skills, including driving vehicles [2], discharging firearms [3,4], defensive tactics [4,5], civilian or partner rescue, vaulting obstacles, and pursuing and apprehending suspects [6,7,8]. Academy training is where law enforcement academy (LEA) instructors and Tactical Strength and Conditioning Facilitators will train recruits to tolerate the physical rigors of the profession, while also teaching the necessary procedures and skills required for the job [9,10].

Physical training is a common practice for most law enforcement academies, as this is used to physically and mentally prepare recruits for their vocation. Many academies tend to focus on muscular endurance or body weight exercises, in addition to aerobic-focused training (e.g., long, slow distance or formation runs), due to large class numbers and limited equipment and space [11,12]. Law enforcement academy staff will often use specific fitness tests to measure these qualities for job retention purposes, or as a reward system (i.e., higher fitness scores could result in the attainment of an award or recognition within the class). Some examples of fitness tests used include muscular fitness or endurance assessments (e.g., maximal push-ups and sit-ups within a certain time period) [1,9,13,14,15,16,17], and running tests over different distances to indirectly measure anaerobic (e.g., 201 m, or 220 yard, run) [16,17] and aerobic (e.g., 2.4 km, or 1.5 mile, run) capacity [1,9,13,14,15,16,17,18].

Many agencies will also have a final examination of job-specific physical skills. This is done to ensure that the recruits will be able to perform the tasks necessary when undertaking police work [6], in order to keep themselves, their colleagues, and the community safe. Within the state of California in the USA, this final examination is referred to as the work sample test battery (WSTB) [19]. Batteries such as the WSTB are typically based on critical job tasks for law enforcement officers. The WSTB consists of five tests completed for time: a run around a 99 yard (90.53 m) obstacle course (99OC); a body drag (BD) with a 165 pound (74.84 kg) dummy; a climb over a six foot chain link fence (CLF); a climb over a six foot solid wall (SW); and a 500 yard (457.2 m) run (500R). These tasks must be completed within a certain time limit, which allows a certain number of points to be assigned to each test [19]. The faster a recruit completes the test, the more points they are allocated [19]. This process also means recruits can essentially “compensate” for poorer performance on one test with a better performance on another. To attain any points, the 99OC must be completed within 33.5 s (sec); the BD within 27.9 s; the CLF within 15.1 s; the SW within 19.6 s; and the 500R within 199.9 s [19]. This means that all the tests within the WSTB are primarily anaerobic in nature [20], which is reflective of the critical tasks for police officers [21]. However, the structure of many LEA academy training programs is not always conducive to the development of anaerobic capacity [11,12], especially as it pertains to maximal strength.

Previous research has documented that extended physical ability tests, where numerous job tasks are performed in succession, relate to performance in physical fitness assessments such as the vertical jump, push-ups, sit-ups, and aerobic endurance in LEOs [6,22]. As previously stated, many law enforcement training academies tend to emphasize circuit training [12] and long, slow distance running [11], operating within a “one-size-fits-all” model [10,11,12]. The term “long, slow distance running” refers to higher volume training completed a constant pace, and this is the recognized definition within the field [23,24]. These training modalities tend to develop muscular endurance and aerobic capacity [9,10], perhaps more so than qualities such as maximal strength. In addition to this, it is also commonplace for LEA training staff to use strength endurance and aerobic capacity assessments to measure fitness in their recruits [13,14,15,16,17,25,26], as these tend to be more easy to implement especially considering time and resource restraints [26]. Thus, how muscular endurance and aerobic capacity might influence the performance of short duration job-specific tasks, such as those within the WSTB, has yet to be determined. The effects of these qualities (or potential lack of influence) is particularly important to note in tasks that might demand maximal or near-maximal strength or muscular force output (e.g., dragging a body or climbing a fence).

Therefore, this study analyzed the relationships between measures of physical fitness with WSTB performance in male and female law enforcement recruits from one LEA. The agency in question utilized a battery of general fitness tests called the PT500 to assess their recruits, and the WSTB as a measure of job-specific task performance. A cross-sectional and retrospective analysis of pre-existing data collected from LEA recruits was conducted. It was hypothesized that there would be significant correlations between the PT500 test elements and the WSTB, and assessments within the PT500 would predict WSTB performance. However, the strength of the correctional and predictive relationships would be dependent on whether a high degree of muscular strength was required for successful task completion.

## 2. Materials and Methods 

### 2.1. Subjects

Retrospective analysis on recruits from four academy classes from one agency was conducted. This convenience sample was comprised of 253 recruits (age: 26.69 ± 5.26 years; height: 1.75 ± 0.10 m; body mass: 79.69 ± 12.29 kg), which included 219 males (age: 26.69 ± 5.35 years; height: 1.77 ± 0.08 m; body mass: 81.94 ± 10.98 kg) and 34 females (age: 26.68 ± 4.68 years; height: 1.62 ± 0.09 m; body mass: 64.43 ± 9.57 kg). The four training cohorts started their academy within a calendar year in southern California. Based on the retrospective nature of this analysis, the institutional ethics committee approved the use of pre-existing data (HSR-17-18-370).

### 2.2. Procedures

The data in this study were collected by staff working for one LEA. The staff were all trained by a certified Tactical Strength and Conditioning Facilitator who verified the proficiency of the staff members. Each recruit’s age, height, and body mass were recorded at the start of the 22-week academy training period, and included here to provide a general description of the characteristics of the recruits. Height was measured barefoot using a portable stadiometer (Seca, Hamburg, Germany), while body mass was recorded by electronic digital scales (Health o Meter, Neosho, MO, USA). The PT500 and WSTB were completed on separate days in the final weeks of academy depending on the class schedule, and typically between 05:00–12:00 (5:00 a.m.–12:00 p.m.). The weather conditions for testing were typical of the climate of southern California during a calendar year. Although conducting testing outdoors is not ideal, there was no indoor testing facility available for this LEA and these procedures were typical of staff from the LEA (i.e., during the hiring process, for recruits during academy, and for incumbents during skill refresher programs). 

### 2.3. PT500

The PT500 was used by staff at this LEA to award fitness pins for high-performing recruits, and was comprised of six assessments: maximal push-ups, sit-ups, and mountain climbers completed in 120 s; maximal number of pull-ups; 201 m run; and 2.4 km run. Rather than the total score for the PT500, the elements of the test were considered individually in this study. The PT500 was an established standard of fitness assessment used by this specific agency for a number of years, and typical of law enforcement recruit tests, incorporated predominantly strength endurance and aerobic assessments [13,14,15,16,17,25,26]. Furthermore, the procedures adopted during testing are reflective of research incorporating law enforcement populations in the published scientific literature [1,6,9,10,13,14,16,17,18,25,26,27,28,29,30,31]. Recruits performed the PT500 in their typical physical training attire. The push-ups, sit-ups, and mountain climbers were conducted outdoors on a concrete surface, and each test was completed with a partner who counted the number of repetitions before the partners alternated. Pull-ups were completed on an outdoor bar. The 201 m and 2.4 km run was performed on an athletics track at the LEA’s facility, and the recruits completed the runs in their platoons, which consisted of 10–15 per group. The procedures for each assessment are detailed hereafter. 

*Push-ups:* Upper-body strength endurance was assessed via a maximal push-up test where recruits completed as many repetitions as possible in 120 s. The technique used for this test is provided here, but has also been presented in great detail in previous literature [1,6,9,13,14,16,17,26,27,28]. Recruits started in the standard “up” position, with the body taut and straight, the hands positioned shoulder-width apart, and the fingers pointed forwards. Staff at the LEA utilized a standard water bottle to determine the bottom position of the push-up, which was positioned underneath the recruit’s chest [18]. On the start command, a LEA staff member began the stopwatch, and candidates flexed their elbows, lowered themselves until their chests contacted the water bottle, before extending their elbows to return to the start position. The recruits performed as many push-ups as possible using this technique. 

*Sit-ups:* Muscular endurance of the abdominal muscles was assessed via the sit-up test, where the recruits completed as many repetitions as possible in 120 s. As for the push-up test, the technique used for this test is provided here, but has also been presented in great detail in previous literature [1,6,9,13,14,16,17,26,27]. The recruits laid on their backs with their knees flexed to 90°, heels flat on the ground, and hands cupped behind their ears. The feet were held to the ground by a partner during the test. On the start command, recruits raised their shoulders from the ground while keeping their hands cupped at their ears and touched their elbows to their knees. The recruit then descended back down until their shoulder blades contacted the ground, and completed as many repetitions as possible.

*Mountain climbers*: This exercise is another muscular endurance assessment. Mountain climbers involve isometric work for the trunk musculature and dynamic hip and knee flexion and extension [32,33]. Recruits started in the standard “up” position for the push-up, and maintained the position with the arms extended throughout the test. The back was required to remain in neutral alignment, and recruits alternated flexing the hip and knee for each leg in movements that brought the knee close to the chest and the foot underneath the body within each repetition. The recruits completed as many repetitions as possible in 120 s.

*Pull-ups:* The pull-up test provided a measure of upper-body pulling strength [34,35], and has been previously used for law enforcement personnel [17,25]. The start position required the recruit to hang on the bar in a vertical position with their hands shoulder-width apart using a pronated grip. The recruit then pulled themselves up while maintaining a vertical body alignment until their chin was over the bar. This counted as one repetition. The recruit then had to descend to a position where the arms were fully extended, and continue until they could no longer get their chin over the bar.

*201 m (220 yard) run*: The 201 m run provided a measure of anaerobic capacity [20], and has been used to assess this capacity in custody assistant recruits [16,17]. The 201 m distance was marked on an athletics track. The recruits were instructed to run the distance as quickly as possible. Time for each recruit was recorded to the nearest 0.1 s by a handheld stopwatch.

*2.4 km run*: The 2.4 km run is a common test used to assess aerobic fitness in law enforcement and tactical populations [1,9,13,14,16,18,26,29,30,31]. The recruits completed six laps around the 400-m track at the training facility and were instructed to run this distance as quickly as possible. The run time was recorded for each recruit on a handheld stopwatch to the nearest 0.1 s. Time was recorded as minutes: seconds (min: s).

### 2.4. Work Sample Test Battery (WSTB)

The WSTB is mandatory for LEAs in California, and recruits must attain a certain standard in order to graduate from academy [19]. A minimum score of 384 is required, and recruits attain points within each test relative to their time to perform each test [19]. The procedures have been presented by Peace Officer Standards and Training [19], but each test has been briefly described here. All tests were performed outdoors on structures specifically designed for the LEA training facility, and recruits wore their physical training attire (i.e., no equipment). The tests were completed in the order presented hereafter, and recruits were provided the opportunity for two attempts for each test (with a minimum of 2 min rest between attempts). Time was recorded to the nearest 0.1 s by a handheld stopwatch for each attempt, and the fastest time was recorded. 

**Figure 1:**
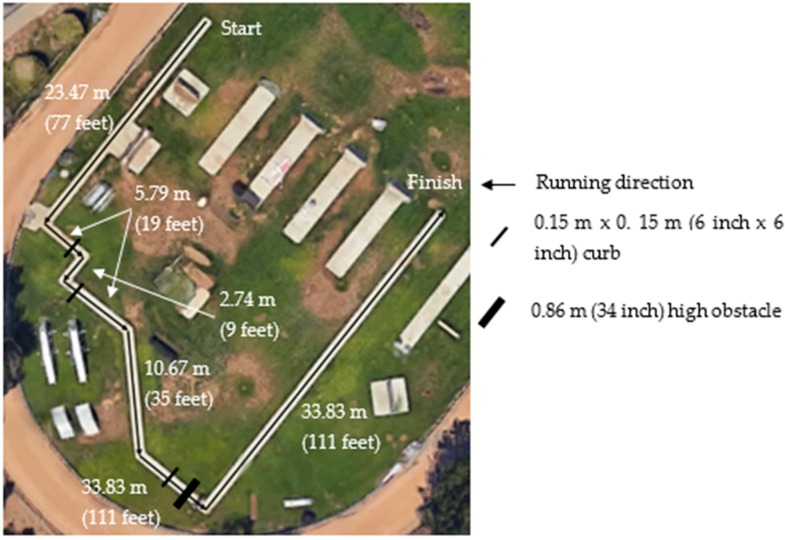
The 99 yard obstacle course.

*99 yard obstacle course run (99OC)*: This test was designed to simulate a foot pursuit, and is shown in Figure 1. Recruits were instructed to complete the 99 yard (90.53 m) course as quickly as possible, while remaining on the concrete track. During the run, they also needed to step over three 6 inch × 6 inch (0.15 × 0.15 m) simulated curbs, and one 34 inch (0.86 m) high obstacle.

*Body drag (BD):* Recruits were required to drag a 165 pound (74.84 kg) dummy a distance of 32 feet (9.75 m). Recruits were required to pick up the dummy by wrapping their arms underneath the arms of the dummy and lifting it to a standing position by extending the hips and knees. Once the recruit was standing with the dummy and they informed the tester they were ready, timing was initiated, and the recruit had to drag the dummy as quickly as possible by walking backwards over the required distance.

*Chain link fence climb (CLF):* Recruits started 5 yards (4.57 m) away from the fence, and once the test was initiated, they were required to run up to and scale the fence with whatever technique they deemed most appropriate. However, they could not use any side supports on the fence to assist their climb. If the recruit did not initially climb the fence in their first attempt within a trial, they could continue attempting to climb, but the time continued to run within the test. Once the recruit cleared the fence, they were to land and run 25 yards (22.86 m) as fast as possible to complete the test.

Solid wall fence climb (SW): The same instructions and procedures for the CLF were provided for the SW, with the only difference being the type of wall that needed to be climbed.

*500 yard run (500R):* This test was designed to simulate a long-distance foot pursuit. The 500 yard (457.20 m) distance was marked on an athletics track, and recruits were instructed to run this distance as quickly as possible.

### 2.5. Statistical Analysis

All statistical analyses were computed using the Statistics Package for Social Sciences (Version 22.0; IBM Corporation, New York, NY, USA). Descriptive statistics (mean ± standard deviation (SD); 95% confidence intervals (CI)) were calculated for each test parameter, and stem-and-leaf plots confirmed a normal distribution in data for each variable [36,37]. Partial correlations controlling for sex were used to determine relationships between the PT500 (push-ups, sit-ups, mountain climbers, pull-ups, 201 m run, and 2.4 km run) with the WSTB (99OC, BD, CLF, SW, and 500R). An alpha level of *p* < 0.05 was required for significance. Partial correlations were used because numerous studies have documented sex differences in the physical performance of law enforcement populations [15,16,38,39], even though there are no corrections for sex in the WSTB [19]. Indeed, previous research has combined data for the sexes in law enforcement research [13,18,26,38,40]. The correlation strength was designated as: an *r* between 0 and ±0.3 was considered small; ±0.31 to ±0.49, moderate; ±0.5 to ±0.69, large; ±0.7 to ±0.89, very large; and ±0.9 to ±1 near perfect for relationship prediction [41]. Stepwise linear regression analyses (*p* < 0.05), with sex as a control variable, were conducted for the WSTB (each test within the WSTB acted as a dependent variable) to illustrate whether a particular PT500 test predicted WSTB performance. This approach was undertaken due to the exploratory nature of this study.

## 3. Results

Descriptive data for the PT500 and WSTB is shown in Table 1. Correlation data between the PT500 and WSTB is shown in Table 2. The 500R correlated to all tests in the PT500; the 99OC correlated with all tests except push-ups and mountain climbers, although significance equalled 0.05 in both cases. There were small-to-moderate correlations with the strength-related tests, and each relationship indicated higher repetitions were associated with a faster time. The 201-m run had small, positive relationships with both the 99OC and 500R. The 2.4 km run had a small relationship with the 99OC, and a large relationship with the 500R. Regarding the CLF, there were small relationships with sit-ups and the 2.4 km run, and a moderate relationship with pull-ups. Greater sit-ups and pull-ups, and a faster 2.4 km run, related to a faster CLF. For the SW, there were small relationships with mountain climbers and the 2.4 km run, and a moderate relationship with pull-ups. Similar to the CLF, these relationships indicated more repetitions in the strength tests, and a faster 2.4 km run time, related to a faster SW. The BD did not significantly correlate with any test in the PT500.

The stepwise linear regression data is shown in Table 3. Pull-ups, push-ups, and the 2.4 km run predicted the 99OC, with an explained variance of 30.1%. Pull-ups and push-ups predicted the CLF (explained variance = 41.8%), while pull-ups predicted the SW (explained variance = 49.3%). The 2.4 km run and pull-ups predicted the 500R with an explained variance of 50.0%. None of the reported tests that comprise the PT500 were predictive of the BD, and the control variable of sex had an *r*^2^ of 0.05 (explained variance = 5.0%).

## 4. Discussion

This study documented the relationships between an agency-specific fitness testing battery called the PT500, with a state-specific job-specific testing battery called the WSTB, in law enforcement recruits from one LEA. The results provided some support to the study hypotheses, and indicated that for all but the BD, there were small-to-moderate correlational and predictive relationships between the PT500 and WSTB. These generally indicated the potential benefits of law enforcement recruits possessing greater muscular endurance, upper-body pulling strength, and anaerobic and aerobic capacity for performing job-specific tasks. However, there is scope for investigating the relationships that maximal strength could have on job-specific task performance, especially when considering the BD.

The 99OC and 500R are both maximal running tests. Both tests had small-to-moderate correlations with the strength tests (push-ups, sit-ups, mountain climbers, and pull-ups) and the 201-m run. All of these PT500 tests place some emphasis on the anaerobic energy system [20], which highlights why there may have been significant relationships with most of these tests and the 99OC and 500R. Further, previous research has indicated that law enforcement recruits [26] and officers [6] who are generally fitter tend to perform well in a range of different fitness and job-specific tests. Due to the test duration, the 500R would also require greater aerobic contribution, which highlights why there was a stronger relationship with the 2.4 km run, both in the correlations and stepwise regression. Indeed, Moreno et al. [42] found a large significant relationship (*r* = −0.61) between 300-m run time and multistage fitness test shuttles in incumbent officers. The results from the current research suggest that law enforcement recruits that have superior muscular endurance, anaerobic capacity, and aerobic capacity could perform better in job-specific tasks and tests such as the 99OC and 500R.

The CLF and SW provide a test of how well a recruit can scale an obstacle, such as that they might encounter in urban areas. Pull-ups significantly correlated and predicted both the CLF and SW. The ability to complete pull-ups provides a measure of upper-body pulling, and to a certain extent, maximal pulling strength [34,35]. Greater upper-body pulling strength could ultimately be beneficial to climbing tasks such as the CLF and SW. Push-ups contributed to the regression for the CLF, and relative pushing ability could be used in the final part of the fence climb for the recruit to elevate their body clear of the fence. The tests of abdominal muscular endurance (i.e., sit-ups and mountain climbers) were also related to performance in the wall climb tests. The abdominal muscles are active during climbing activities [43,44], and this should include tasks such as the CL and SW. Aerobic capacity as measured by the 2.4 km run was also related to the CLF and SW, but this may be a function of fitter recruits tending to be better in all measures of physical capacity [26]. Even so, the current data indicated that greater upper-body strength and abdominal muscular endurance are qualities that may positively influence job-specific tasks such as the CL and SW.

What should be noted is that although the physical fitness qualities measured in the PT500 related to and predicted the 99OC, 500R, SW, and CLF, there are still other contributing factors to the performance of these tests. For example, there are technical elements to each of the WSTB tasks. Linear sprinting [45,46] and change-of-direction [47,48] technique will greatly influence running speed and the ability to maneuver around corners and obstacles. Any form of climbing requires appropriate technique [44]. Although technique was not measured in this study, and is not generally considered by LEA training staff as long as recruits complete each test in the required time [19], it would likely impact on any correlational or predictive analyses. Perhaps more notably as it pertains to fitness testing, maximal strength could also influence performance in sprinting and single-effort climbing tasks [49]. Maximal lower-body strength was not measured in the PT500; this is potentially even more impactful when considering the possible correlates for the BD.

The BD did not have any significant relationships with the PT500 fitness tests, nor was predicted by any of them. The BD in the WSTB simulates a victim drag or civilian rescue type scenario, which is often assessed in LEOs [7,8]. It could be expected that lower-body strength should contribute to this task, given the need for the recruit to pick up the dummy from the ground and extend the legs to move to a standing position [19]. Dawes et al. [6] suggest that dynamic strength may be a better predictor for physical ability tests in LEOs, especially when compared to other strength measures (e.g., isometric strength). It is important to note that high-intensity or high-load strength training may not always be a focus of academy training [11,12]. As such, if recruits do not develop their strength and ability to generate high levels of muscular force, this could impact the performance of a task such as the BD. This supposition is supported by the introduction of a 220 pound (99.79 kg) hexagonal bar deadlift by the US Army as a strength metric to assess incoming recruits, with the assessment based on the strength needed for a casualty evacuation [50]. This type of strength test could be explored in law enforcement recruits as well. Strength measured in this manner could relate to the performance of the BD, which is especially important due to the relatively heavier body mass, compared to previous years, that currently exists in the general population for males and females [51]. 

There are certain study limitations to this study that should be noted. This study incorporated a pre-existing fitness testing battery specific to one agency. Different LEA staff may use different fitness assessments (e.g., the multistage fitness test instead of the 2.4 km run) [6,15,42,52], which could influence the relationships demonstrated with job-specific tests. No high-intensity (maximal or near-maximal force output) strength tests were included in the PT500. The strength of law enforcement recruits, and how this may impact job-specific task performance, requires further investigation. Further to this, the WSTB is only mandatory in the state of California in the USA. Other states and countries may use different job-specific testing protocols, and these should be analyzed specifically for each agency, state, or country. Nonetheless, within the context of these limitations, this is the first known study to analyze general fitness tests with job-specific task performance as measured by the WSTB in law enforcement recruits. 

## 5. Conclusions

In conclusion, select relationships between physical fitness, as measured by the PT500, and job-specific performance as measured by the WSTB were identified. Muscular endurance, and anaerobic and aerobic capacity could influence running tasks such as the 99OC and 500R. Upper-body strength, especially pulling strength, in addition to abdominal strength, may influence climbing tasks (SW and CLF). Development of these qualities may not just influence the WSTB, but job-specific task performance as well. Accordingly, training staff from a LEA should ensure their recruits have the requisite muscular endurance, anaerobic capacity, and aerobic capacity to successfully complete the WSTB. Development of these qualities could then also crossover into occupational law enforcement tasks. However, the BD was not related to or predicted by any test in the PT500. This may be because maximal lower-body strength is more important to a task that simulates dragging a person. The influence of maximal strength on job-specific task performance requires further exploration in law enforcement recruits, and should be a focus of future research.

## Figures and Tables

**Table 1:** Descriptive data for the PT500 (a battery of general fitness tests) and the work sample test battery (WSTB) in law enforcement recruits (*n* = 253).

Variables	Mean ± SD
PT500	
Push-ups (repetitions)	55.66 ± 12.20
Sit-ups (repetitions)	65.28 ± 11.64
Mountain Climbers (repetitions)	58.67 ± 8.49
Pull-ups (repetitions)	13.86 ± 6.70
201 m Run (s)	32.21 ± 17.59
2.4 km Run (min: s)	11:10 ± 1:14
WSTB	
99OC (s)	18.49 ± 1.63
BD (s)	5.41 ± 3.19
CLF (s)	7.83 ± 1.20
SW (s)	7.75 ± 1.37
500R (s)	89.20 ± 7.99

99OC: 99 yard obstacle course run; BD: body drag with a 165 lb dummy; CLF: climb over a six foot chain link fence; SW: climb over a six foot solid wall; 500R: 500 yard run.

**Table 2:** Relationships between the PT500 and WSTB in law enforcement recruits (*n* = 253).

		99OC	BD	CLF	SW	500R
Push-ups	*r*	0.125	0.024	0.094	−0.053	−0.128 *
	*p*	0.050	0.710	0.143	0.412	0.045
Sit-ups	*r*	−0.208 *	−0.001	−0.175 *	−0.203	−0.344 *
	*p*	0.001	0.989	0.006	0.001	<0.001
Mountain Climbers	*r*	−0.126	0.049	−0.020	−0.127 *	−0.264 *
	*p*	0.050	0.441	0.757	0.048	<0.001
Pull-ups	*r*	−0.272 *	−0.024	−0.315 *	−0.309 *	−0.372 *
	*p*	<0.001	0.710	<0.001	<0.001	<0.001
201-m Run	*r*	0.127 *	0.002	0.037	0.106	0.140 *
	*p*	0.046	0.971	0.561	0.096	0.029
2.4 km Run	*r*	0.253 *	0.011	0.131 *	0.190 *	0.574 *
	*p*	<0.001	0.861	0.041	0.003	<0.001

* Significant (*p* < 0.05) relationship between the two variables.

**Table 3:** Stepwise linear regression analysis between the WSTB and PT500 in law enforcement recruits (*n* = 253).

Variables	*r*	*r* ^2^	Significance
99OC			
Pull-ups	0.466 *	0.217	<0.001
Pull-ups, Push-ups	0.526 **	0.276	<0.001
Pull-ups, Push-ups, 2.4 km Run	0.549 **	0.301	<0.001
CLF			
Pull-ups	0.613 **	0.376	<0.001
Pull-ups, Push-ups	0.647 **	0.418	<0.001
SW			
Pull-ups	0.702 ***	0.493	<0.001
500R			
2.4 km Run	0.698 **	0.487	<0.001
2.4 km Run, Pull-ups	0.707 ***	0.500	<0.001

NOTE: Sex was used as a control variable and so was involved in all significant relationships; only those physical fitness tests that predicted the WSTB are noted here. * Moderate, ** Large, *** Very Large.
